# A case of successful endoscopic full-thickness resection for removal of a metal spring buckle penetrating the gastric antral wall

**DOI:** 10.1055/a-2814-5944

**Published:** 2026-03-16

**Authors:** Cuimei Ma, Yaowen Zhang, Zongjing Hu, Shuqing Zhu, Dehuai Jing

**Affiliations:** 1 562122 Department of Gastroenterology, Affiliated Hospital of Jining Medical University, Jining, China; 2 562122 Endoscopy Department, Affiliated Hospital of Jining Medical University, Jining, China

**Fig. 1:**
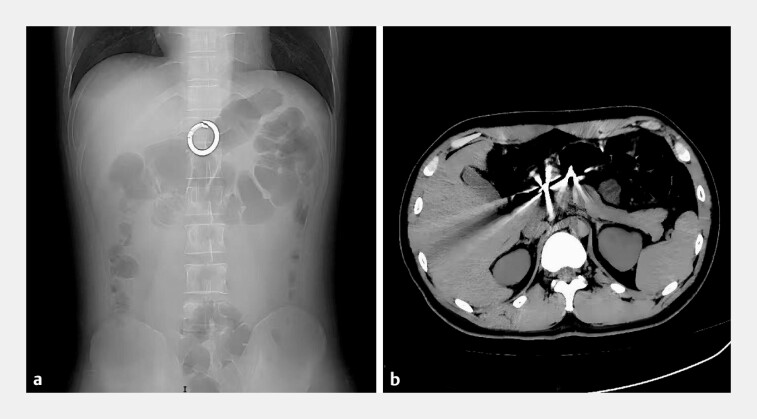
**a** The X-ray reveals a circular foreign object in the upper abdomen. **b** The computed tomography suggests a circular foreign body embedded in the gastric antral wall with no clear signs of gastrointestinal perforation.

**Fig. 2:**
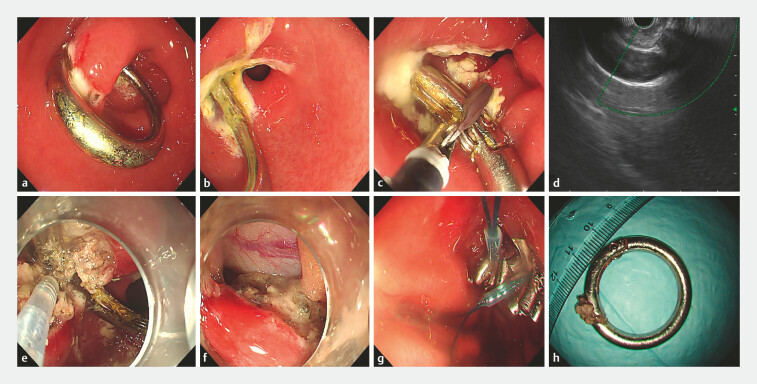
**a** A circular metallic foreign body is embedded within the gastric antral wall. **b** Erosion and ulcers are observed on the gastric mucosa at both ends of the impacted foreign body. **c** Attempted removal of the foreign body using rat-toothed forceps. **d** An endoscopic ultrasound shows no significant large blood vessels around the impacted foreign body. **e** Gradual dissection of the metal ring using a dual knife combined with an IT2 knife. **f** Blurred boundaries between the buckle portion of the metal ring and the muscularis propria of the stomach, with the resulting wound after full-thickness gastric wall incision. **g** Closure of the wound using metal clips combined with a nylon loop. **h** The retrieved circular metal spring buckle, approximately 3.6 cm in diameter.

A 26-year-old male patient with depression was admitted after intentionally swallowing a circular metal buckle for 2 days. The patient shows no signs of peritonitis. Abdominal X-ray and computed tomography indicated a foreign body in the gastric antrum ( Fig. 1 **a, b** ). Gastroscopy showed a circular metallic foreign body embedded in the gastric wall at the antrum. Erosions and ulcers were observed in the gastric mucosa surrounding the foreign body ( Fig. 2 **a, b** ). Multiple attempts to remove the foreign body with rat-tooth forceps revealed that its both ends were fully transmurally embedded, resulting in unsuccessful extraction ( Fig. 2 **c** ). Endoscopic ultrasonography showed no significant large vessels surrounding the impacted foreign body ( Fig. 2 **d** ). Therefore, we performed an endoscopic full-thickness resection ( Video 1 ).

A case of successful endoscopic full-thickness resection for removal of a metal spring buckle penetrating the gastric antral wall.Video 1

A dual knife was used to perform a full-thickness incision of the gastric wall along the line connecting the two ends of the metal ring. Due to indistinct boundaries between the metal ring buckle and muscularis propria, a combination of the dual knife and IT2 knife was employed for dissection, ultimately achieving complete liberation of the metal ring. The foreign body had caused a penetrating injury to the gastric wall. Metal clips combined with a nylon loop were applied for effective closure. Subsequently, a gastric tube was inserted, and the foreign body was successfully extracted using rat-tooth forceps ( Fig. 2 **e–h** ). The patient was kept on nil per os for 72 hours postoperatively and received antibiotic prophylaxis along with proton and pump inhibitor therapy. Three days later, he began oral water intake without significant discomfort. He recovered smoothly and was discharged 1 week later. At 1-month follow-up after operation, the patient reported no significant discomfort.

Foreign bodies that penetrate the gastric wall and retained for a prolonged period are prone to cause gastric perforation or fistula formation 1 . If endoscopic retrieval fails, surgical intervention is typically required 2 . In this case, the foreign body was successfully extracted using endoscopic techniques, thereby avoiding more invasive surgical procedures.

Endoscopy_UCTN_Code_TTT_1AO_2AG_3AF Endoscopy_UCTN_Code_TTT_1AO_2AO
